# Three-Year Clinical Follow-Up of Children Intrauterine Exposed to Zika Virus

**DOI:** 10.3390/v13030523

**Published:** 2021-03-22

**Authors:** Rosa Estela Gazeta, Ana Paula Antunes Pascalicchio Bertozzi, Rita de Cássia de Aguirre Bernardes Dezena, Andrea Cristina Botelho Silva, Thamirys Cosmo Gillo Fajardo, Daniel T. Catalan, Maria de Fátima Valente Rizzo, Antonio Fernandes Moron, Antoni Soriano-Arandes, Nuria Sanchez Clemente, Tania Quintella, Dora Fix Ventura, Francisco Max Damico, Valtenice de Cassia Rodrigues de Matos França, Juliana Paula Gomes de Almeida, Ana Laura de Sene Amâncio Zara, Lucas Castro Pires, Cohort Zika vírus Jundiaí, Saulo Duarte Passos

**Affiliations:** 1 Pediatrics Department, Jundiaí Medical School, Jundiaí 13202-550, Brazil; bertozzianap@gmail.com (A.P.A.P.B.); fatrizzo@hotmail.com (M.d.F.V.R.); sauloduarte@uol.com.br (S.D.P.); 2 University Center Campo Limpo Paulista-UNIFACCAMP, Campo Limpo Paulista, São Paulo 13231-230, Brazil; rita10nurse@yahoo.com.br; 3 Pediatric Infectology Laboratory, Jundiaí Medical School, Jundiaí 13202-550, Brazil; andreabotelhobm@gmail.com (A.C.B.S.); thamirysfajardo@hotmail.com (T.C.G.F.); dtcatalan@hotmail.com (D.T.C.); 4 Tropical Medicine Institute, Campus São Paulo, University of São Paulo (USP), São Paulo 05403-000, Brazil; antonio_moron@uol.com.br; 5 Paediatric Infectious Diseases and Immunodeficiencies Unit, Hospital Universitari Vall d’Hebron, Vall d’Hebron Research Institute, Universitat Autònoma de Barcelona, 08035 Barcelona, Spain; asoriano@vhebron.net; 6 Infectious Disease Epidemiology, London School of Hygiene and Tropical Medicine, London WC1E 7HT, UK; nuriasanchezclemente@gmail.com; 7 Pediatric Emergency Department, Pediatrics Department -Pontifícia Universidade Católica de Campinas, Campinas 13086900, Brazil; tqtatella@gmail.com; 8 Department of Experimental Psychology, Institute of Psychology, Campus São Paulo, University of São Paulo, São Paulo 05588-030, Brazil; dventura@usp.br (D.F.V.); fmdamico@yahoo.com (F.M.D.); valtenice@yahoo.com.br (V.d.C.R.d.M.F.); 9 Pediatric Neurology Department, School of Medical Sciences of Santa Casa de São Paulo, São Paulo 01221-010, Brazil; julianapga@hotmail.com; 10 Department of Collective Health, Tropical InstitutePathology and Public Health, Federal University of Goiás, Goiânia 74690-000, Brazil; analauraufg@gmail.com; 11 Faculty of Medicine, Jundiaí Medical School, Jundiaí 13202-550, Brazil; lucas.cpires@hotmail.com; 12 Cohort Zika vírus Jundiaí, Jundiaí 13207-450, Brazil; adm.projetozika@gmail.com

**Keywords:** Zika virus, congenital infection, microcephaly, cohort studies

## Abstract

Congenital Zika virus (ZIKV) infection may present with a broad spectrum of clinical manifestations. Some sequelae, particularly neurodevelopmental problems, may have a later onset. We conducted a prospective cohort study of 799 high-risk pregnant women who were followed up until delivery. Eighty-three women and/or newborns were considered ZIKV exposed and/or infected. Laboratory diagnosis was made by polymerase chain reaction in the pregnant mothers and their respective newborns, as well as Dengue virus, Chikungunya virus, and ZIKV serology. Serology for toxoplasmosis, rubella, cytomegalovirus, herpes simplex virus, and syphilis infections were also performed in microcephalic newborns. The newborns included in the study were followed up until their third birthday. Developmental delay was observed in nine patients (13.2%): mild cognitive delay in three patients, speech delay in three patients, autism spectrum disorder in two patients, and severe neurological abnormalities in one microcephalic patient; sensorineural hearing loss, three patients and dysphagia, six patients. Microcephaly due to ZIKV occurred in three patients (3.6%). Clinical manifestations can appear after the first year of life in children infected/exposed to ZIKV, emphasizing the need for long-term follow-up.

## 1. Introduction

Since Gregg uncovered the correlation between rubella infection during pregnancy and newborn congenital defects [1] innumerable studies have been conducted to establish the mechanisms behind feto–maternal transmission of congenital infections [2]. Although careful follow-up of these affected children is crucial, more studies to explore the longer-term manifestations of these congenital infections are needed. The same phenomenon occurred with Zika virus (ZIKV). The correlation between ZIKV pregnancy infection and microcephaly in the newborn made the Brazilian Ministry of Health (BMH) declare, in 2015, a Public Health Emergency of National Concern, and later on, a Public Health Emergency of International Concern was declared by the World Health Organization (WHO) [3].

In March 2016, Brazilian researchers made the association between vertical ZIKV transmission and fetal abnormalities, such as microcephaly, cerebral calcifications, agenesis or abnormalities of the corpus callosum, ocular abnormalities, and arthrogriposis, among others [4,5]. In December 2016 many South, Central and North American countries described congenital Zika syndrome (CZS) cases, with a total of 2525 confirmed cases, from which 2289 (90%) were in Brazil [6].

According to BMH, from November 2015 to October 2019, there were 18,282 probable CZS cases, and from January to June 2020, there were 2054 probable CZS cases (incidence rate of 1.0 case/100,000 inhabitants). Although the numbers are decreasing, the risk of a new emergence of the ZIKV must be taken into account [7].

Clinical manifestations of CZS have a broad spectrum and a variable intensity. The congenital infection during the first trimester often causes severe fetal abnormalities, particularly in the central nervous and cardiovascular systems, in addition to ocular, auditory, and osteoarticular impairments [8,9]. The real extent of the neurological impairment still remains to be determined. Studies performed in regions of high incidence of ZIKV show that even the newborn head circumference (HC) is normal at birth; yet, these newborns can present long-term developmental cognitive, adaptive, and behavioral abnormalities [10,11]. These data suggest that the extension of these sequelae may be greater than it was thought at the beginning of the ZIKV epidemic [12].

The need for a multiprofessional team to follow-up these affected children generates a high economic and social burden to the countries in which ZIKV is endemic, requiring more studies on this disease [10]. The longitudinal follow-up is important to understand the spectrum of ZIKV infection and its repercussions on childhood. Considering this fact, the objective of the present study was to identify the clinical abnormalities that present in a group of children exposed to ZIKV during pregnancy, from birth up to three years of age.

## 2. Materials and Methods

The present study is a nested-cohort study, part of the Zika Project Jundiaí previously published [13]. This study was performed at the University Hospital of Jundiaí (UHJ), in Brazil, from 1st March 2016 to 30th June 2019. UHJ is a public hospital that receives patients from Jundiai and six neighboring cities. During this period of time, 799 children were followed-up and divided into two different groups.

Maternal cohort: Comprised by the high-risk pregnant mothers, which were invited to participate in the cohort and were followed-up on a monthly basis. Their follow-up consisted of clinical examination, gestational ultrasound, and biologic material sampling (blood, saliva, and urine) to perform Zika virus RT-qPCR and serology (blood). The prenatal follow-up was considered optimal according to the Kotelchuch index [14]. From this maternal cohort, 694 newborns were evaluable for our study.

Nested-cohort: During the period of the study, 105 children with suspected microcephaly were included in the project, and this cohort was denominated the nested-cohort. The mothers of those children were not included in the study.

Incomplete data and microcephaly due to infectious causes other than Zika virus were excluded from the study. The control group was composed of RT-PCR children with negative serologies for ZIKV and other congenital infections, while their mothers presented negative tests for ZIKAV, dengue virus, and chikungunya (Image 1).

At birth, the anthropometric measures (weight, length, and head circumference) (W, L, HC) of the newborns were taken by members of the research team previously trained. Clinical follow-up was carried out according to WHO guidelines [15]. Gestational age was calculated according to obstetric ultrasound up to the 20th week of pregnancy and when this was not available, the last menstrual period (LMP) was used. Gestational age by the Capurro method [16] was used only in cases where the others parameters were not adequate. Microcephaly was considered when the newborn HC was equal or less than 2 standard deviations (SD) under the mean measurement expected for sex and gestational age considering the standard curve INTERGROWTH-21st for both term and preterm babies [17]. Physical examination of the newborn was carried out during the first 24 h, according to a specific protocol (attachment 1).

According to the BMH, CZS is every newborn who presents, at any gestational age, a positive result for ZIKV up to the 8th day of postnatal life, TORCHS (toxoplasmosis, rubella, cytomegalovirus, herpes simplex virus, and syphilis) negative or inconclusive, and one or more signs or symptoms compatible with ZIKV infection (clinical or image) [18].

All included children were followed-up on a monthly basis during the first year of life, and then at 14, 16, 24, 30, and 36 months of age, following protocols stratified by age, with expected vaccination status, clinical, anthropometric, and developmental data (attachment 2).

The subgroup of ZIKV exposed children and/or microcephalic were followed clinically by the same protocol, and were additionally examined by a group of neurologists, physical therapists, speech therapists, psychologists, and ophthalmologists.

Visual acuity was evaluated by the theTeller AcuityCards II (TAC II; Stereo Optical Co, Chicago, IL, in collaboration with Vistech Consultants Inc, Dayton, OH), with distance adapted for each age [19], as well as indirect binocular ophthalmoscopy (ID-5 BIO, Topcon, Tokyo, Japan) at 2, 6, and 12 months of age.

The auditory acuity was evaluated by optoacoustic emissions (OAE) in all the newborns, and for those ZIKV exposed, brain evoked response audiometry (BERA) and imitanciometry were performed at birth, 6, 12, and 24 months of age. 

Cognitive and motor developmental evaluation were performed in the microcephalic and ZIKV-exposed children, as well as in the children with detected developmental delay, through the Bayley scale, third edition (BSIDIII, Pearson Assessments, Brazil) [20], validated for the Brazilian population, at 2, 4, 6, 12, 24, and 36 months of age. For preterm newborns, the postnatal age was corrected according to the gestational age at birth [21]. 

Dyphagia was evaluated in the ZIKV exposed patients and in patients in which suggestive symptoms were present, using the Montreal Children’s Hospital Feeding Scale [22,23]. Genetic evaluation was performed in all microcephalic children, ZIKV exposed or not.

Data of the children lost to follow-up were actively searched in the outpatient public services of Jundiaí and its region. In some cases, these data could not be retrieved. 

As a result of a considerable number of losses of follow-ups, the analysis of the variable was made by using the last available evaluation of the patient. Microcephaly and osteoarticular abnormalities data used were those obtained at birth, independently of follow-up, according to the study protocol. The data used to write this article can be found at https://coortejundiai.lightning.force.com/lightning/page/home (accessed on 5 February 2021).

**Figure 1:**
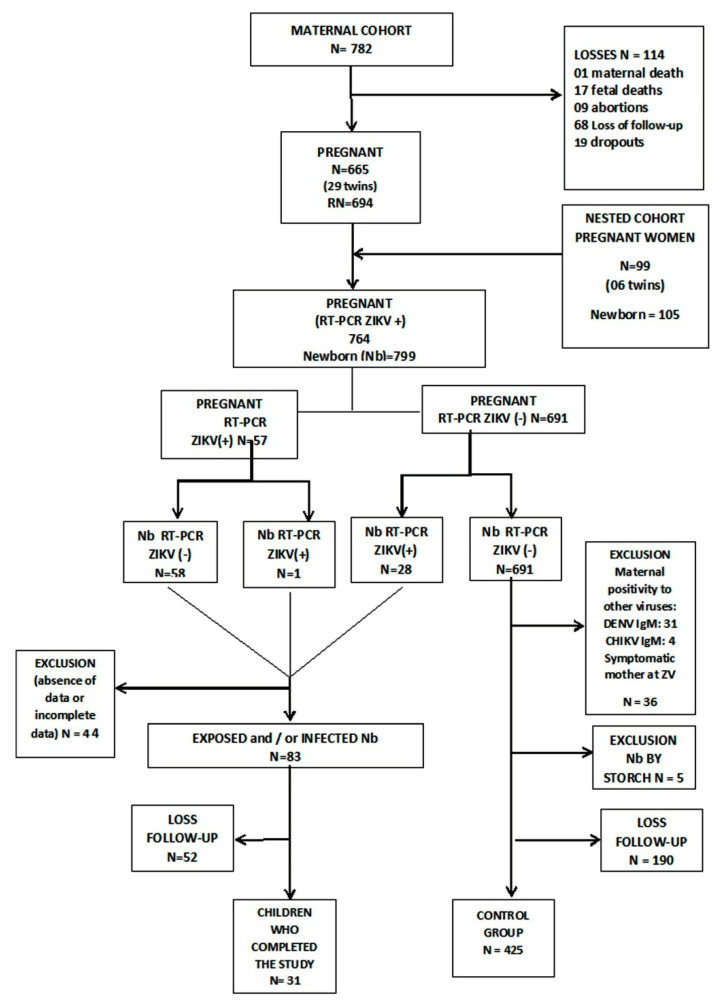
Cohorts’ composition, children groups, and losses of the study.

Figure 1 shows the cohort composition, children groups, and losses to follow up.

### 2.1. Laboratory Diagnosis

Blood, urine, and saliva samples were collected from the pregnant mothers monthly, during prenatal follow-up, and ZIKV quantitative real-time polymerase chain reaction (RT-qPCR), was made according to Lanciotti et al. [24], using Creative Biogene Zika Virus (ZIKV) Real Time RT-PCR Kit. The blood samples were also analyzed by enzyme-linked immunosorbent assay (ELISA) to detect IgM and IgG antibodies for dengue, Chikungunya, and ZIKV, using Euroimmune commercial kit.

In the newborn group, blood, saliva and urine were collected to perform RT-qPCR for ZIKV. The newborn was considered “infected” when RT-qPCR was positive within the first eight days of postnatal life. 

Cerebrospinal fluid and blood of the exposed newborns were also collected to exclude possible TORCHS infection. 

### 2.2. Statistical Analysis

All collected data were registered in a duplicate manner and were analyzed using Statistical Package of Social Sciences (IBM SPSS Statistics 22, IBM Corporation, Armonk, NY, USA). Qualitative variables were expressed in simple and relative frequencies. Quantitative variables were presented in means, standard deviations, and medians (first and third quartiles, and minimal and maximum values). To compare clinical characteristics between exposed and/or positive children with the control group, we used Pearson chi-squared test, Fisher, and/or Mann–Whitney tests. The adopted significance level was 0.05 (α = 5%). In order to identify risk factors, we calculated the odds ratio, with a confidence interval of 95% (95% CI) and performed multivariate and univariate logistic regression.

### 2.3. Ethical Procedures

All pregnant women included in the study signed the informed consent form (ICF) after explanation, reading, and clarification of the study, its objectives, and procedures. The underage patients (under 18 years old) signed an agreement term and a legal responsible signed an ICF. 

The study was approved in the Ethical Committee of the Jundiaí Medical School, approval number 1446577, and it was executed according to ethical stablished standards (2013 Helsinki Declaration and Brazilian National Health Resolution of 12 December 2012).

## 3. Results

### Cohort General Characteristics

Table 1 describes the sociodemographic, clinical, and epidemiological data of the cohort high-risk pregnant women.

Regarding ZIKV infection, 57 (7.5%) pregnant women were positive by the RT-qPCR method. 

In this group, 31 (4%) positive cases for dengue IgM and 4 cases (0.5%) of dengue and ZIKV co-infection were observed. From these co-infected women, one of them presented acute infection symptoms during the gestational period and the respective newborn presented clinical findings compatible with ZIKV infection. The attachment 3 describes these data. 

According to WHO criteria, 40 (5.3%) pregnant women were considered positive for acute ZIKV infection. In this study, the presence of symptoms in the pregnant mother did not correspond to ZIKV congenital infection in the newborn (*p* value > 0.05). 

From these 57 pregnant mothers, we had a sample of 59 newborns (2 cases of twins), which were considered “exposed”. From these children, three were excluded due to missing data. Another group, comprising 28 supplementary newborns whose mothers were Zikv negative, presented as Zikv RT-qPCR positive at birth. This group of children were considered “positive”. From this group, one child was excluded due to missing data. 

The clinical and laboratorial findings of these groups can be observed in the attachments 3 and 4. 

The birth anthropometric data are described in Table 2.

When we compare the three different groups, we can observe that the exposed and the positive newborns presented a significatly higher birth weight (OR = 0.35; CI 95%0.16–0.79; *p* = 0008). Due to a significant number of pregnant women with diabetes mellitus I and II and gestational diabetes, the comparison of the birth weight among the three groups of newborns was made in a separate way (newborns of diabetic and non-diabetic mothers). Still, there was a significant difference between the control group and the two other groups (OR = 0.44; CI 95% 0.18–0.97; *p* = 0046) (Table 3).

Using the same approach, birth weight was compared between the exposed/positive group and control group according to gestational age (term and preterm). The frequency of low-birth weight was significantly higher in the group control when compared to the exposed/positive newborn groups, independent of the gestational age (Table 4).

When we consider the microcephaly cases, we can observe that ZIKV infection was detected by RT-qPCR in three cases and one case (1.7%) in an exposed newborn. The characteristics of these newborns can be observed in attachments 3 and 4. The other cases of microcephaly were: 22 (45.8%) newborns diagnosed with intrauterine growth restriction (IUGR), 15 (31.2%) preterm and with IUGR, three (6.2%) with genetic syndromes, and three (6.2%) with cytomegalovirus infection. 

Clinical and radiographic findings compatible with congenital ZIKV were separately analyzed and are described in Table 5. Other findings of maternal dengue and Chikungunya are also explained in this table. In the cohort, we did not find arthrogriposis or muscle contractures suggestive of ZIKV infection. 

Among the exposed children, the risk of developmental abnormalities was 3.85 times greater than among the children of the control group (OR = 3.85; 95% CI1.65–8.96; *p* = 0003). Dysphagia was also more frequent among the ZIKV-exposed group (OR = 3.68; 95% CI 1.26–10.76; *p* = 0.022).

Five children presented radiological findings consistent with ZIKV congenital infection; three out of five also presented clinical manifestations (attachments 3 and 4). 

If we consider the BMH definition for CZS during the period of 2016 to 2020, only one patient (0.1%) of the study could be diagnosed. Changing to the CDC criteria, four children (0.5%) presented with CZS. When we analyzed the exposed/positive children with developmental abnormalities, we found a mild cognitive delay in three cases, speech delay in three cases, autism spectrum disorder (ASD) in two cases, and one case of severe development impairment (CZS) (attachments 4 and 5). 

Table 6 presents factors associated with ZIKV infection. After multivariate analysis, the most important factors were dengue positivity in the pregnant mother (OR = 4.13; 95% IC 1.04–16.42) and developmental abnormalities in the children (OR = 10.33; 95% IC 1.96–54.50).

It is important to address that there were a considerable number of children lost to follow-up, especially in the first months of the study, as demonstrated in Figure 1. We had only 31 exposed/positive children with complete data at 36 months of age.

## 4. Discussion

Microcephaly has been the most important alert sign associated with CZS since the beginning of the epidemic in 2015 [7,18] and its report has been mandatory in Brazil since then. Apart from microcephaly, severe brain abnormalities may be present after ZIKV infection, especially in the first weeks of pregnancy [25]. The incidence of microcephaly related to intrauterine ZIKV infection was very different depending on the region of study and the population studied. In Latin American countries, it varied from 0.3 to 14.3% [26] whereas, in the Brazilian states most affected by ZIKV, this incidence varied between 5.6 and 45% in the period between 2015 and 2017 [27,28]. Of the 48 cases of microcephaly diagnosed in the cohort, only 3 (3.6%) cases were related to ZIKV infection (one of them in a child with CZS), a percentage that is similar to those of other regions with a low incidence of congenital infections by ZIKV Brady. The reasons for the different rates of microcephaly related to ZIKV are not yet clear and some hypotheses will be discussed later on in this paper. The other microcephaly cases found in the cohort were related to Cytomegalovirus infection, genetic syndromes, and most of them to prematurity and IUGR. The last factor can be explained as most of the pregnant mothers had chronic or pregnancy-related diseases [21].

When we analyzed the birth weight, exposed/positive newborns presented a significantly higher weight in comparison to the control group. Low birth weight is a common finding in congenital infections, despite the etiology, and it is often associated with the gestational age and the severity of the infection [27]. In CZS, IUGR is also important and the weight gain in postnatal life can be markedly low for months, and even years [29]. In our cohort, both facts may have occurred, and we need more studies to better address this result.

Hearing impairment can occur in ZIKV-exposed children, but it is not a frequent manifestation. In a study made in Pernambuco in 2015, from 104 microcephalic children positive for ZIKV infection, hearing impairment was detected only in the two (12%) with the most severe microcephaly case [30]. Sensorineural hearing loss was described in another study, with 5 out 70 ZIKV-exposed children detected [31]. The data are conflicting. A study conducted in Recife involving 78 ZIKV-exposed children (symptomatic and asymptomatic) demonstrated sensorineural hearing loss of 5.1% (four cases, one asymptomatic) [32]. On the other hand, Barbosa et al., in a study with 29 ZIKV-exposed children, did not find hearing impairments [33]. In our cohort, hearing impairments were observed in asymptomatic or mild symptomatic cases. 

Language disorders are frequently described in children with CZS and are a consequence of severe CNS impairment, craniofacial changes, and sensorineural hearing loss, among other factors [34]. There are, however, few reports in the literature on children exposed to intrauterine ZIKV infection without microcephaly. In a Puerto Rico case-control study with a group of 29 ZIKV-exposed children without microcephaly or CZS and 36 controls, a significant higher delay in receptive language was observed in the exposed group [35]. Speech delay is a common event of multifactorial cause, affecting up to 15% of normal children over 2 years of age. In about half of the cases it can have a spontaneous resolution, but for a significant number of children, speech therapy intervention may be necessary. Untreated cases may evolve to a restricted vocabulary at the end of speech acquisition, causing future disorders in learning and communication [36]. Nevertheless, further studies are needed to clarify whether there is a relationship between speech delay and intrauterine exposure to the Zika virus.

Autistic spectrum disorder has a complex pathophysiological basis and is related to the interaction of genetic, environmental, and infectious factors, among which are congenital infections, such as congenital rubella syndrome [37]. Higher rates of autism occur in children with hearing, visual, and intellectual disabilities. Congenital ZIKV infections have a high probability of presenting ASD due to the cerebral inflammatory process that is established, influencing the process of neurogenesis and neurodevelopment [38]. There are few reports of autism and ZIKV infection. A study in Rio de Janeiro involving 216 children exposed to intrauterine ZIKV detected, among other neurological changes, three cases of ASD in children with normal development until the second year of life [32]. The data found in our cohort need more detailed investigations so that we can relate them to congenital Zika virus infection.

Dysphagia can be present in CZS and, in some cases, alternative ways of feeding are necessary due to aspiration and malnutrition in the first months of life [38,39]. Nevertheless, dysphagia has already been described in ZIKV-exposed patients without severe neurological manifestations. In a cohort study made in 2017, 58 children exposed to ZIKV without microcephaly or other neurological manifestations and 58 with CZS were analyzed. Of the 58 children without microcephaly, 53 (91.4%) did not have dysphagia, 3 (5.2%) had mild oropharyngeal dysphagia, and 2 (3.4%) had moderate to severe DOF. In contrast, the group of children with microcephaly had 12 cases (20.7%) without dysphagia, and 12 (20.7%) and 34 (58.4%) cases with mild and moderate to severe dysphagia, respectively [39]. We still need more studies to confirm the causal association between OFD and ZIKV infection/exposition in these children.

In this work, we verified the occurrence of few cases with early clinical manifestations, such as microcephaly and CZS, with the late manifestations prevailing, normally related to sensorineural hearing loss and neuropsychomotor development. The incidence and severity of the impairment of children exposed to gestational ZIKV varies significantly according to the study location and the selection criteria of the studied population [40,41,42]. In general, the most serious cases occurred in poor areas with fewer health resources, which usually present a great proliferation of *Aedes aegypti*, thus increasing the risk of exposure to ZIKV and, consequently, to other arboviruses, especially dengue [43]. There is a high risk of viral co-infection in such areas, whose consequences are not yet fully elucidated, but which can have serious outcomes. The clinical manifestations in viral coinfection can vary from asymptomatic forms due to the inhibition of action among the pathogens involved, to severe forms due to the potentiation of the action of these agents [44,45,46,47]. Additionally, in low-income regions, there is a high risk of chronic malnutrition in pregnant women and poor nutritional status can increase the adverse effects of ZIKV infection [47]. Nevertheless, Jundiaí has the seventh greater gross domestic product (GDP) of the Sate of São Paulo, in Brazil.

Finally, it is important to emphasize the losses of follow-up in the present study. This is a frequent issue that can compromise the final results of many cohort studies. We had a loss of 65.6% of the exposed/positive patients, mostly in the first six months of life. Some studies indicate losses of follow-ups varying from 56 to 64%, with low socioeconomic income, low level of education, difficulty in access to health services, and low familiar support being the main causes to these losses [48,49,50]. In this cohort, all of these factors could have contributed to our losses. Although we cannot prove this, we strongly suspect that one of the reasons for this losses in our cohort may have been the fact that, once the newborn did not present suggestive abnormalities associated with CZS at birth, the mothers abandoned the study. Despite the losses of follow-up, we were able to follow the cases of exposed and/or infected children by comparing them to the control group, which brought relevant findings.

## 5. Conclusions

ZIKV congenital infection still presents many aspects that must be studied, especially the spectrum of clinical manifestations. Most of the exposed children were asymptomatic and, among the symptomatic group, late neurological manifestations were the most common finding, which may have a greater dimension than expected at the beginning of this epidemic. 

Microcephaly was not significant in our study, and this finding shows the need for a broader clinical investigation, in order to find other clinical signs of ZIKV infection. This is the reason why these exposed children must be clinically followed for a longer period, with special attention to developmental and neurological evaluation. 

In our study, the fact that we included high-risk pregnant women may be a limitation factor that may have possibly interfered in the final result. The loss of follow-up of a great number of children may also have some implications in the comprehension of the true dimension of the clinical spectrum of these ZIKV-exposed children.

## Figures and Tables

**Table 1:** Sociodemographic, clinical, and epidemiological data of the cohort high-risk pregnant women, 2016–2019.

Sociodemographic Characteristics	Pregnant Women (*n* = 764)
Age (years) (*n* = 763)	
Mean (±standard deviation)	27.4 (±7.3)
Median (IIQ 25–75%)	27 (21–33)
Minimum–maximum	13–46
Age (years)	n (%)
13 to 19	123 (16.1)
20 to 34	497 (65.1)
35 to 46	143 (18.7)
Not informed	1 (0.1)
Ethnicity	
White	398 (52.1)
Brown	267 (34.9)
Black	79 (10.3)
Indigenous	2 (0.3)
Not informed	18 (2.4)
Paid work	
Yes	346 (45.3)
No	401 (52.5)
Not informed	17 (2.2)
Risk factors	
Gestational or type I diabetes mellitus	199 (26)
Chronic or gestational arterial hypertension	78 (10.2)
Early Pregnancy	71 (9.3)
Microcephaly (suspected or confirmed)	50 (6.5)
Twinning	25 (3.3)
Suggestive symptoms Zika1	11 (1.4)
Others	274 (35.8)
No information	14 (2.6)
Changes in obstetric ultrasound compatible with Zika	
Yes	41 (5.4)
No	713 (93.3)
Not informed	10 (1.3)
Type of delivery	
Vaginal	358 (46.9)
Cesarean	371 (48.6)
Forceps	18 (2.4)
No information	16 (2.1)
Zika symptoms	
Yes	40 (5.2)
No	723 (94.7)
Not informed	1 (0.1)
Zika Virus PCR	
Detectable	57 (7.5)
Not detectable	691 (90.4)
No information	16 (2.1)
Dengue (IgM)	
Detectable	31 (4.0)
Not detectable	624 (8.,7)
No information	109 (14.3)
Chikungunya (IgM)	
Detectable	4 (0.5)
Not detectable	731 (95.7)
No information	29 (3.8)
Viral co-infection	
Zika and dengue	4 (0.5)

**Table 2:** Anthropometric characteristics of the exposed, positive, and control newborns at birth, Jundiaí Medical School, 2016–2019.

Clinical Characteristics	Total	Children with Zika or Exposed (Mothers Confirmed with Zika)	Control (Non-Reactive)	*p*-Value	OR (95% CI)
Gestational age (weeks) [n (%)]	*n* = 797	*n* = 83	*n* = 672		
≤31	21 (2.6)	1 (1.2)	20 (3.0)	0.496 ^1^	0.38 (0.05–2.88)
32 to 36	114 (14.3)	10 (12.1)	104 (15.5)	0.376 ^2^	0.73 (0.37–1.47)
≥37	620 (77.8)	72 (86.7)	548 (81.5)	-	1
Weight (grams)	*n* = 777	*n* = 82	*n* = 695		
Mean (±standard deviation)	2970.9 (±636.3)	3147.4 (±549.3)	2950.1 (±643.0)	-	-
Median (IIQ 25–75%)	3015 (2618–3398)	3222 (2790–3526)	2980 (2595–3375)	0.006 ^3^	-
Minimum–maximum	560–4525	1270–4285	560–4525	-	-
Low weight at birth	*n* = 83	*n* = 672			
Yes (<2.500 g)	151 (19.4)	7 (8.5)	144 (20.7)	0.008 ^2^	0.35 (0.16–0.79)
No (≥2.500 g)	626 (80.6)	75 (91.5)	551 (79.3)	-	1
Length (cm)	*n* = 82	*n* = 692			
Mean (± standard deviation)	47.4 (±3.3)	48.3 (±2.7)	47.3 (±3.3)	-	-
Median (IIQ 25–75%)	48 (46–50)	48 (47–50)	48 (46–50)	0.004 ^3^	-
Minimum–maximum	28.5–54.5	38.0–53.0	28.5–54.5	-	-
APGAR 5 min	*n* = 695	*n* = 78	*n* = 617		
Mean (±standard deviation)	9.3 (±0.8)	9.3 (±0.8)	9.3 (±0.8)	0.692 ^3^	-
Median (IIQ 25–75%)	9 (9–10)	9 (9–10)	9 (9–10)	-	-
Minimum–maximum	4–10	5–10	4–10	-	-
APGAR 5 min	*n* = 695	*n* = 83	*n* = 672		
1 a 6	8 (1.2)	1 (1.3)	7 (1.1)	1.000 ^1^	1.13 (0.14–9.32)
7 a 10 (normal)	687 (98.8)	77 (98.7)	610 (98.9)	-	1
Head circumference (cm)	*n* = 768	*n* = 82	*n* = 686		
Mean (±standard deviation)	33.6 (±2.3)	34.1 (±1.9)	33.6 (±2.3)	-	-
Median (IIQ 25–75%)	34 (33–35)	34 (33–35)	34 (32–35)	0.163 ^3^	-
Minimum–maximum	21–38.5	27–38	21–38.5	-	-
Microcephaly [n (%)]	*n* = 797	*n* = 83	*n* = 694		
Yes	48 (6.0)	3 (3.6)	45 (6.5)	0.295 ^2^	0.53 (0.16–1.76)
No	730 (91.6)	81 (96.4)	649 (93.5)		1

^1^ Fisher’s exact test (*p* < 0.05). ^2^ Chi-square test (*p* < 0.05). ^3^ Mann-Whitney U test (*p* < 0.05).

**Table 3:** Association of birth weight between positive/exposed newborns and control newborns according to maternal diabetes, Junidaí Medical School, 2016–2019.

Clinical Characteristics	Total	Children with Zika or Exposed (Mothers Confirmed with Zika)	Control (Non-Reactive)	*p*-Value	OR (95% CI)
Pregnant women with diabetes mellitus ^1^					
Weight (grams)	*n* = 206	*n* = 28	*n* = 178		
Mean (±standard deviation)	3206.1 (±532.1)	3280.0 (±403.7)	3194.5 (±549.6)	-	-
Median (IIQ 25–75%)	3250 (2890–3555)	3250 (2950–3599)	3250 (2960–3598)	0.582 ^2^	-
Minimum–maximum	720–4330	2560–4090	720–4330	-	-
Low weight at birth					
Yes (<2.500 g)	14 (6.8)	-	14 (100.0)	0.224 ^3^	0.20 (0.11–3.43)
No (≥2.500 g)	192 (93.2)	28 (14.6)	164 (85.4)	-	1
Pregnant women without diabetes mellitus					
Weight (grams)	*n* = 553	*n* = 53	*n* = 500		
Mean (±standard deviation)	2877.9 (±642.7)	3074.1 (±608.2)	2857.1 (±654.4)	-	-
Median (IIQ 25–75%)	2930 (2515–3330)	3115 (2680–3495)	2920 (2486–3281)	**0.020 ^2^**	-
Minimum–maximum	560–4525	1270–4285	560–4525	-	-
Low weight at birth					
Yes (<2.500 g)	135 (24.4)	7 (5.2)	128 (94.8)	**0.046 ^4^**	0.44 (0.18–0.97)
No (≥2.500 g)	418 (75.6)	46 (11.0)	372 (89.0)	-	1

^1^ Type I, type II and gestational diabetes mellitus; ^2^ Mann-Whitney U test (*p* < 0.05); ^3^ Fisher’s exact test (*p* < 0.05); ^4^ Chi-square test (*p* < 0.05); Values in bold indicate statistically significant differences.

**Table 4:** Comparison of low birth weight and gestational age in the newborn groups (exposed/positive) versus control, Jundiaí Medical School, 2016–2019.

Clinical Characteristics	Total	Children with Zika or Exposed (Mothers Confirmed with Zika)	Control (Non-Reactive)	*p*-Value	OR (IC95%)
Preterm birth ^1^					
Weight (grams)		*n* = 11	*n* = 120		
Mean (±standard deviation)	2293.4 (±712.1)	2337.3 (±551.4)	2289.4 (±726.8)	-	-
Median (IIQ 25–75%)	2320 (1840–2865)	2370 (2200–2695)	2305 (1825–2876)	0.845 ^3^	-
Minimum–maximum	560–3573	1270–3055	560–3573	-	-
Low weight at birth					
Yes (<2.500 g)	76 (58.0)	6 (7.9)	70 (92.1)	>0.999 ^4^	0.86 (0.25–2.97)
No (≥2.500 g)	55 (42.0)	5 (9.1)	50 (90.9)	-	1
Term birth ^2^					
Weight (grams)	*n* = 615	*n* = 70	*n* = 545		
Mean (±standard deviation)	3119.0 (±513.7)	3269.2 (±434.0)	3099.7 (±520.2)	-	-
Median (IIQ 25−75%)	3120 (2780–3475)	3272 (2924–3555)	3090 (2760–3455)	0.009 ^3^	-
Minimum–maximum	1320–4525	2340–4285	1320–4525	-	-
Low weight at birth					
Yes (<2.500 g)	69 (11.2)	1 (1.4)	68 (98.6)	0.006 ^5^	0.10 (0.01–0.74)
No (≥2.500 g)	546 (88.8)	69 (12.6)	477 (87.4)	-	1

^1^ Preterm birth: less than 37 weeks; ^2^ term birth: 37 weeks or more; ^3^ Mann-Whitney U test (*p* < 0.05); ^4^ Fisher’s exact test (*p* < 0.05); ^5^ Chi-square test (*p* < 0.05).

**Table 5:** Clinical and laboratory findings of the ZIKV-exposed children, Jundiaí Medical School, 2016–2019.

Variables		Total	Children with Zika or Exposed (Mothers Confirmed with Zika)	Control (Non-Reactive)	*p*-Value	OR (IC95%)
Result of dengue in pregnant women (exposure)						
	Positive	34 (4.3)	8 (10.5)	26 (4.3)	0.043 ^1^	2.62 (1.14–6.03)
	Negative	648 (81.3)	68 (89.5)	580 (95.7)	-	1
	No information	115 (14.4)	-	-	-	-
Result of chikungunya in pregnant women (exposure)						
	Positive (IgM)	4 (0.5)	1 (1.2)	3 (0.4)	0.369 ^1^	2.76 (0.28–26.84)
	Negative	761 (95.5)	82 (98.8)	679 (99.6)	-	1
	No information	32 (4.0)	-	-	-	-
Vision impairment						
	Yes	8(1)	1	7 (3.0)	0.353 ^1^	0.27 (0.01–4835.00) ^2^
	No	281 (35.3)	55 (100.0)	226 (97.0)	-	1
	No information	509 (63.8)	-	-	-	-
Arthrogryposis						
	Yes	-	-	-	-	-
	No	572 (71.8)	65 (100.0)	507 (100.0)	-	1
	No information	225 (28.2)	-	-	-	-
Hearing Loss						
	Yes	8 (1.0)	3 (4.9)	4 (1.5)	0.123 ^1^	3.40 (0.74–15.61)
	No	312 (39.1)	58 (95.1)	263 (98.5)	-	1
	No information	479 (59.9)	-	-	-	-
Abnormalities in post-natal imaging						
	Yes	17 (2.1)	5 (11.1)	12 (13.0)	0.747 ^2^	0.83 (0.28–2.53)
	No	120 (15.1)	40 (88.9)	80 (87.0)	-	1
	No information	660 (82.8)	-	-	-	-
Abnormalities of neuropsychomotor development						
	Yes	27 (3.4)	9 (13.2)	18 (3.8)	0.003 ^1^	3.85 (1.65–8.96)
	No	513 (64.4)	59 (86.8)	454 (96.2)	-	1
	No information	257 (32.2)	-	-	-	-
Dysphagia						
	Yes	15 (1.9)	6 (10.0)	9 (2.9)	0.022 ^1^	3.68 (1.26–10.76)
	No	352 (44.2)	54 (90.0)	298 (97.1)	-	1
	No information	429 (53.9)	-	-	-	-

^1^ Fischer’s Exact Test (*p* < 0.05). Added 0.5 to each cell to make the OR calculation possible (Openepi). ^2^ Chi-square test (*p* < 0.05).

**Table 6:** Multivariate analysis of the factors associated with ZIKV infection in the newborns of the cohort, Jundiaí medical School, 2016–2019.

Variables	Univariate Analysis OR (95% CI)	Multivariate Analysis OR (95% CI)
**Positive dengue result in pregnant women**	**2.62 (1.14–6.03)**	**4.13 (1.04–16.42)**
Hearing Loss	3.40 (0.74–15.61)	1.29 (0.12–13.57)
**Abnormalities of neuropsychomotor development**	**3.85 (1.65–8.96)**	**10.33 (1.96–54.50)**
**Dysphagia**	**3.68 (1.26–10.76)**	**1.99 (0.38–10.45)**
Low weight at birth	0.35 (0.16–0.79)	0.54 (0.15–2.01)
Microcephaly	0.46 (0.18–1.17)	0.28 (0.06–1.22)

Bold values indicate an association with a diagnosis of Zika in children who are confirmed or exposed to the virus during pregnancy.

## Data Availability

Not applicable.

## Supplementary Materials

The following are available online at https://www.mdpi.com/1999-4915/13/3/523/s1. Attachment 1: Follow-up newborn, infant and child microcephaly protocol/zika exposure; Attachment 2: Follow-up medical check; Attachment 3: Clinical Manifestations of Children exposed to ZIKV; Attachment 4: Clinical manifestations of children positive to ZIKV.
