# Supplementary material for: Three-Year Clinical Follow-Up of Children Intrauterine Exposed to Zika Virus
Source: Viruses. 2021 Mar 22;13(3):523. doi: 10.3390/v13030523 (PMC8005078; doi:10.3390/v13030523)
Supplement: Supplementary file 1 [file viruses-13-00523-s001.zip › Attachment 1_Follow-up Newborn infant and child microcephaly protocol _ Zika Exposure.docx]

**Attachment 1**

**FOLLOW-UP NEWBORN, INFANT AND CHILD MICROCEPHALY PROTOCOL / ZIKA EXPOSURE**

Date of follow-up: _______/_________/______ Birth date _______/_________/______

Mother´s name: __________________________________________________________________________________

Child name: _____________________________________________________________________________________

Address: ________________________________________________________________________________________

Telephone: ___________________ Best time for contact: ( ) Morning ( ) Afternoon ( ) Evening

**INFANT / NEWBORN DATA:**

Delivery : ( )0. vaginal ( ) 1.Cesaran section ( ) 2.forceps ( ) 999.non-referred / unknown

- Gestational Age

| **Based on:** | **☐** LMP (last menstrual period)  **☐**Ultrasound  **☐** Assisted reprodutive technology  **☐**Other (specify) |
| --- | --- |

| **Birth data** | **☐** Singleton **☐** Twin (1^st^) **☐**  Twin (2^nd^)  **☐**other: ______ |
| --- | --- |

- Birth weight_______ Birth length__________ Birth head circumference________
- Apgar score 5 min_______
- Labor complications: ( )0.no ( ) 1.yes ( )999. non-referred / unknown
- If yes, describe:______________________________________________________
- Perinatal events: ( ) No ( ) Yes If yes, describe: _______________________________________________________________________

| **Maximum temperature** | | ____.__°C  **☐**Oral **☐☐** Ear **☐☐** Anal **☐☐**Axillary  **☐**Another (specify): | | | | | |
| --- | --- | --- | --- | --- | --- | --- | --- |
| **Respiratory rate** | |  | | | | ipm | |
| **Heart Rate** | |  | | | | bpm | |
| **Capillary filling time** | |  | | | | seconds | |
| **Peripheral oxygen saturation** | |  | | | | % | |
| **Cardiovascular system** | **☐**Normal  **☐**Abnormal  **☐**Unknown | If abnormal, describe | | | | | |
| **Sistema respiratório** | **☐**Normal  **☐**Abnormal  **☐** Unknown | If abnormal, describe | | | | | |
| **Gastrointestinal system** | **☐**Normal  **☐**Abnormal  **☐** Unknown | **☐** jaundice **☐** abdominal pain **☐**Hepatomegaly **☐** Splenomegaly  **☐** Other (specify) : | | | | | |
| **Crying type** | ☐ normal **☐** weak  **☐** absent **☐** Other (specify) : | | | | | | |
| **Asymmetrical tonic neck reflex** | **☐**Present **☐a**bsent  **☐** not done | | **Moro Reflex** | | **☐**Present **☐**Absent  **☐**Not done | | |
| **Search Reflex** | **☐**Present **☐**Absent  **☐**Not done | | **Sucking reflex** | | **☐**Present **☐**Absent  **☐**Not done | | |
| **Grasp reflex** | **☐**Present **☐**Absent  **☐**Not done | |  | |  | | |
| **Seizures** | **☐**Generalized **☐**Focal **☐**absent **☐unknown** | | | | If yes, describe: | | |
| **Paralysis** | **☐**General **☐**Ascendent **☐**absent  ☐ unknown | | | | If yes, describe: | | |
| **Hypotonia** | ☐Yes ☐No ☐Unknown | | | | | | |
| **Hypertonia or Spasticity** | ☐Yes ☐No ☐Unknown | | | If yes, describe: | | | |
| **Contractures** | ☐Yes ☐No ☐Unknown | | | If yes, describe: | | | |
| **Other neurologic signs** | ☐Yes ☐No | | | If yes, describe: | | | |
| **Other movements** | ☐Yes ☐No | | | If yes, describe: | | | |
| **Rash** | ☐Yes ☐ No ☐Unknown If yes, describe: | | | If yes, onset date of skin rash (DD/MM/YYYY) | | | **__ __ /__ __ / 20 __** |
| **Edema** | ☐Yes ☐No ☐Unknown | | | If yes, describe: | | | |

**PHYSICAL EXAMINATION (UP TO 24 H):**

**Birth abnormalities (up to 24 h**)

| **Fontanelles** | Anterior: ☐Yes ☐No  ☐ unknown | Posterior:  ☐Yes ☐No  ☐unknown | Bulging:  ☐Yes ☐No  ☐unknown |
| --- | --- | --- | --- |
| **Cephalohematoma** | ☐ Yes ☐No  ☐unknown | Subgaleal Hemorrhage | ☐Yes ☐No  ☐Unknown |
| **Craniosinostosis** | ☐Yes ☐No  ☐unknown  If yes, describe: | Omphalocele | ☐Yes ☐No  ☐Unknown |
| **Skull skin excess** | ☐Present  ☐Absent  If yes, describe: | Prominent occiput | ☐ Present  ☐ Absent |
| **Down Syndrome characteristics** | ☐Yes ☐No  ☐ unknown | Neural tube defects | ☐yes ☐No  ☐ unknown |
| **Facial dismorphism** | ☐yes ☐No  ☐ unknown  If yes, describe: | Cleft palate / lip | ☐ Yes ☐No  ☐unknown |
| **Eye abnormalities** | ☐ Yes ☐No  ☐unknown  If yes, describe: | Ear abnormalities | ☐ Anotia/microtia  ☐ Other (describe):  ______________________  ☐ No  ☐ Unknown |
| **Hemangiomas** | ☐ Present  ☐ Absent | ☐ Facial  ☐ Other location | Number : ___________  Location:__________ |
| **Congenital cardiopathies** | ☐ Yes ☐No  ☐ unknown | If yes, describe |  |
| **Gastroschisis** | ☐Sim ☐Não  ☐Desconhecido | Hernia umbilical | ☐Yes ☐No  ☐Unknown |
| **Hand abmormalities** | ☐ Polydactyly  ☐ Absence of one or more fingers  ☐ Absent  ☐ Unknown  ☐ Other (specify): | Foot abnormalities | ☐ abnormal width between toes  ☐ clubfoot  _______________________  ☐ No  ☐ Unknown  ☐ Other (specificy): |
| **Artrogriposis** | ☐Yes ☐No  ☐Unknown | If yes, describe: |  |
| **Superior / inferior limbs abnormalities** | ☐Yes ☐No  ☐ Unknown | If yes, describe:  Describe which member |  |
| **Any Other abnormality / importante findings** | ☐Yes ☐No  ☐Desconhecido | If yes, describe |  |

**FAMILY HISTORY**

- Genetic syndromes ( ) no ( ) yes Describe: ____________________
- Microcephaly ( ) no ( ) yes
- Neurologic diseases ( ) no ( ) yes Describe_____________________
- Other____________________________________________________

**LABORATORY EXAMS**

| Date (dd/mm/ano): | __ / __ / 20 __ | | | |
| --- | --- | --- | --- | --- |
| Test | result | Unity | | |
| Reactive C protein |  | mg/L | | other:_____ |
| Erythrocyte sedimentation rate |  | mm | | other:_____ |
| Procalcitonine |  | ng/mL | | other:_____ |
| Hemoglobine |  | g/L | g/dL | other:_____ |
| Hematocrite |  | % | | other:_____ |
| Lekocytes |  | x10^9^/L | x10^3^/µL | other:_____ |
| Neutrophils |  | 10^3^/mm^3^ | % | other:_____ |
| Lymphocytes |  | 10^3^/mm^3^ | % | other:_____ |
| Monocytes |  | 10^3^/mm^3^ | % | other:_____ |
| Eosinophils |  | 10^3^/mm^3^ | % | other:_____ |
| Basophils |  | 10^3^/mm^3^ | % | other:_____ |
| MCV |  | μm^3^ | | other:_____ |
| Erithrocytes |  | x10^9^/L *or* | x10^3^/μL | other:_____ |
| Platelets |  | x10^9^/L *or* | x10^3^/μL | other:_____ |
| aPTT |  | Seconds | | |
| PT |  | Seconds | | |
| BUN |  | mmol/L | mg/dL | other:_____ |
| Albumin |  | g/L | | other:_____ |
| Sodium |  | mEq/L | | other:_____ |
| Potassium |  | mEq/L | | other:_____ |
| Calcium |  | mmol/L | | other:_____ |
| Phosphorus |  | mg/dL | | other:_____ |
| Magnesium |  | mmol/L | | other:_____ |
| Total protein |  | g/dL | | other:_____ |
| Creatinine |  | μmol/L | mg/dL | other:_____ |
| Glucose, serum |  | mmol/L | mg/dL | other:_____ |
| Amylase |  | U/L | | other:_____ |
| Bilirubin |  | µmol/L | mg/dL | other:_____ |
| AST |  | U/L | | other:_____ |
| ALT |  | U/L | | other:_____ |
| Alkalyne phosphatasis |  | U/L | | other:_____ |
| GGT |  | U/L | | other:_____ |
| CK |  | U/L | | other:_____ |
| Other (specificy): |  | Unit:_________ | | |
| Other (specify): |  | Unit:_________ | | |
| Blood smear | Yes Not done Unknown | | | |
| If yes, describe |  | | | |

**Ear / Eye Tests**

| **Test** | **Results** | **If abnormal, describe:** |
| --- | --- | --- |
| **Fundoscopy** | **☐**Normal **☐**Abnormal **☐**Not done |  |
| **Red reflex test** | **☐**Present **☐**Absent **☐**Not done |  |
| **Cataract** | **☐**Normal **☐**Abnormal **☐** Not done |  |
| **Chorioretinitis** | **☐**Present **☐**Absent **☐** Not done |  |
| **Hearing test (specify the test performed)** | **☐**Normal **☐**Abnormal **☐** Not done |  |

**IMAGES**

**(IF ABNORMAL, DESCRIBE)**

| **Neuroimage** | **ResultS** |  | | | **Attached image** | **Attached report** |
| --- | --- | --- | --- | --- | --- | --- |
|  |  | **Type of the image** | **Location** | **size** |  |  |
| **Brain ultrasound** | **☐**Normal **☐**Abnormal  **☐**not done |  |  |  | **☐ yes**  **☐ no** | **☐yes**  **☐**No |
| **Brain CT scan** | **☐**Normal **☐**Abnormal  **☐**not done |  |  |  | **☐yes**  **☐**No | **☐ yes**  **☐**No |
| **MRI** | **☐**Normal **☐**Abnormal  **☐**not done |  |  |  | **☐ Yes**  **☐**No | **☐ Yes**  **☐**No |
| **Other (specify):** | **☐**Normal **☐**Abnormal  **☐**not done |  |  |  | **☐ yes**  **☐**No | **☐yes**  **☐**No |

**SEROLOGY AND PCR**

| **Test** | **Was this test done?** | **Date** *(dd/mm/YYYY)* | **Results** |
| --- | --- | --- | --- |
| RT-PCR ZIKA:  - blood  - Urine  - Placenta | yes No  yes No  yes No | I__I__I/I__I__I/I__I__I__I__I  I__I__I/I__I__I/I__I__I__I__I  I__I__I/I__I__I/I__I__I__I__I | Positive Negative  Positive Negative  Positive Negative |
| ZIKA Serology | Yes No | I__I__I/I__I__I/I__I__I__I__I | IgM Pos Neg unknown |
|  |  |  | IgG Pos Neg unknown |
| Dengue serology | Yes No | I__I__I/I__I__I/I__I__I__I__I | IgM Pos Neg unknown |
|  |  |  | IgG Pos Neg unknown |
| Toxoplasmosis | Yes No | I__I__I/I__I__I/I__I__I__I__I | Positive Negative |
| Rubella | Yes No | I__I__I/I__I__I/I__I__I__I__I | Positive Negative |
| Cytomegalovirus | Yes No | I__I__I/I__I__I/I__I__I__I__I | Positive Negative |
| Syphilis | Yes No | I__I__I/I__I__I/I__I__I__I__I | Positive Negative |
| Herpes Simplex | Yes No | I__I__I/I__I__I/I__I__I__I__I | Positive Negative |
| Other (specificy) | Yes No | I__I__I/I__I__I/I__I__I__I__I | Positive Negative |

Final Diagnostics_________________________________________________________________

Medical conduct _________________________________________________________________

____________________________________________

Name and signature
