# Supplementary material for: Three-Year Clinical Follow-Up of Children Intrauterine Exposed to Zika Virus
Source: Viruses. 2021 Mar 22;13(3):523. doi: 10.3390/v13030523 (PMC8005078; doi:10.3390/v13030523)
Supplement: Supplementary file 1 [file viruses-13-00523-s001.zip › Attachment 2_follow-up Medical check.docx]

**Attachment 2**

**FOLLOW-UP MEDICAL CHECK**

Name: __________________________________________________________ Age (days): ___________

Date (DD/MM/YYYY): ______/_____/________.

Microcephaly - At birth ( ) Yes ( ) No

Secondary ( ) Yes ( ) No – Date of the diagnosis: ______/_____/________.

1. **EVENTS**:

__________________________________________________________________________________________________________________________________________________________________________________________________

__________________________________________________________________________________________________________________________________________________________________________________________________

1. **MEDICATIONS**: Vitamin D ( ) Iron sulphate ( ) other

__________________________________________________________________________________________________________________________________________________________________________________________________

1. **FEEDING PATTERN:**
2. Breastfeeding ( ) yes ( ) No
3. Formula milk ( ) yes ( ) No Type of milk:_________________________________________________________
4. Juice / Fruits ( ) yes ( ) No
5. Baby food / pap/ porridge ( ) yes ( ) No
6. Family food ( ) Yes ( ) No
7. Vegetables, meat, egg, milk intake:( ) normal ( ) abnormal

Errors: _____________________________________________________________________________________

1. **IMUNIZATION ACCORDING TO AGE:**

( ) normal ( ) abnormal

If abnomral, describe:_______________________________________________________________________________

1. **NEONATAL SCREENING:**

Date _______________ Normal ( ) abnormal ( )

| 1. **Sensorial DEVELOPMENT** | | | |
| --- | --- | --- | --- |
| Reacts to sound | ☐Yes ☐No | Follows objects | ☐ yes ☐ No |
| Other | ☐ Yes ☐No | If abnormal, describe: | |

If abnormal, describe ________________________________________________________________________________

1. **DEVELOPMENT EVALUATION**

| Smiles after stimulus | ☐ Yes ☐ No | Opens her/his hands | ☐ yes ☐ No |
| --- | --- | --- | --- |
| Vocalizes | ☐ Yes ☐ No | Plays with his/her hands | ☐ yes ☐ No |
| laughs | ☐ Yes ☐ No | Grasps objects | ☐ yes ☐ No |
| Moves Arms When orine | ☐ Yes ☐ No | Grasps objects spontaneously | ☐ yes ☐ No |
| Lifts head When prone | ☐ Yes ☐ No | keeps head sustained | ☐ yes ☐ No |
| Seats with assistance | ☐ Yes ☐ No | SIts without support | ☐ Yes ☐ No |
| Seats unassisted | ☐ Yes ☐ No | Gives objects | ☐ Yes ☐ No |
| Points with his/her finger | ☐ Yes ☐ No | Pincer grasp | ☐ Yes ☐ No |
| Puts a cube in a box | ☐ Yes ☐ No | Crawls | ☐ Yes ☐ No |
| Extend arms to be picked up | ☐ Yes ☐ No | Throws objects | ☐ Yes ☐ No |
| Reacts to his/ her name | ☐ Yes ☐ No | Crawls | ☐ Yes ☐ No |
| Orients to voice and to her/ his name | ☐ Yes ☐ No | Stands up | ☐ Yes ☐ No |
| Comprehends an order | ☐ Yes ☐ No | Walks | ☐ Yes ☐ No |
| Throws a ball | ☐ Yes ☐ No | Walks up/ down | ☐ Yes ☐ No |
| Helps to take off his/her clothes | ☐ Yes ☐ No | Speaks syllables | ☐ Yes ☐ No |
| Undress without help | ☐ Yes ☐ No | First words | ☐ Sim ☐ No |
| Dress unassisted | ☐ Yes ☐ No | Speaks short phrases | ☐ Yes ☐ No |
| Climbs furniture | ☐ Yes ☐ No | Able to speak 4 words in a phrase | ☐ Yes ☐ No |
| Runs | ☐ Yes ☐ No | Names objects | ☐ Yes ☐ No |
| Draws a line | ☐ Yes ☐ No | Eats independently | ☐ Yes ☐ No |
| Opens covers | ☐ Yes ☐ No | Eats correctly | ☐ Yes ☐ No |
| Kicks a ball | ☐ Yes ☐ No | Turns the pages of a book | ☐ Yes ☐ No |
| Climbs stairs | ☐ Yes ☐ No | Plays with home activities (to cook, to sweep, etc) | ☐ Yes ☐ No |
| Daytime urinary control | ☐ Yes ☐ No | Imitates gestures | ☐ Yes ☐ No |
| Points correctly 7 parts of the body | ☐ Yes ☐ No | Stadns tall in one foot | ☐ Yes ☐ No |
| Jumps with both feet | ☐ Yes ☐ No | Holds a pencil correctly | ☐ Yes ☐ No |
| Turns the pages of a book | ☐ Yes ☐ No | Recognizes him/herself at the mirror | ☐ Yes ☐ No |
| Understands movements up/ down | ☐ Yes ☐ No | Identifies his/her gender | ☐ Yes ☐ No |
| Knows his name (complete) | ☐ Yes ☐ No | Other abnormalities | ☐ Yes ☐ No |

1. **NEUROLOGICAL EVALUATION**

| Tipo of crying: ☐ normal ☐ weak or continuous ☐ absent ☐ Other (specificy): | | | | | |
| --- | --- | --- | --- | --- | --- |
| Tonic reflex | ☐ Present ☐ Absent | | Moro Reflex | | ☐ Present ☐ Absent |
| Rooting reflex | ☐ Present ☐ Absent | | Suck reflex | | ☐ Present ☐ Absent |
| Grasp reflex | ☐ Present ☐ Absent | | Plantar grasp | | ☐ Present ☐ Absent |
| Axial Tonus | ☐ yes ☐ No | | Symmetrical mobility | | ☐ yes ☐ No |
| Peripheral tonus | ☐ yes ☐ No | | Hypotonia | | ☐ yes ☐ No |
| Troublesleeping | ☐ yes ☐ No | | Hyperactivity | | ☐ yes ☐ No |
| Neurodevelopmental problems | ☐ yes ☐ No | | Agressivity | | ☐ yes ☐ No |
| Superior limb spasticity | ☐ yes ☐ No | | Inferior limb spasticity | | ☐ yes ☐ No |
| Ataxia | ☐ yes ☐ No | | Suprabulbar syndrome | | ☐ yes ☐ No |
| Dysmetria | ☐ yes ☐ No | | Dystonia | | ☐ yes ☐ No |
| Oculomotor apraxia | ☐ yes ☐ No | | Choreatetosis | | ☐ yes ☐ No |
| Cranial nerves alterations | ☐ yes ☐ No | | If yes, describe: _____________________________________. | | |
| Epilepsy | ☐ Yes ☐ No | | If yes, start date : *I__I__I/I__I__I/I__I__I__I__I* | | |
| Seizures: ☐ West syndrome ☐ Lennox ☐ Febrile ☐ Generalized ☐ Focal | | | | | |
| Atiepileptic drugs | | ☐ Yes ☐ No | | If yes, specificy: _______________________. | |
| EEG (attach results)  ☐ yes ☐ No | |  | | | |
| Other abnormalities:  ☐ Yes ☐ No | | If abnormal, specify: | | | |

1. **PHYSICAL EXAMINATION**

Weight:____________

Percentile: _________

Z-score: ___________

Length: ___________

Percentile: ________

Z-score: __________

BMI: ___________

Percentile: _______

Z-score: ________

H C:_____________

Percentile: ________

Z-score: __________

**General:**

1. Mucosae: ( ) normal ( ) abnormal
2. Hydratio status: ( ) normal ( ) abnormal
3. Cyanosis: ( ) normal ( ) abnormal
4. Jaundice: ( ) normal ( ) abnormal
5. Respiratory rate: ( ) normal ( ) abnormal
6. Peripheral perfusion: ( ) normal ( ) abnormal
7. **Head and Neck:**

Oropharynx: ( ) normal/adequado ( ) abnormal

Ears: ( ) normal/adequado ( ) abnormal

Lymphnodes: ( ) normal/adequado ( ) abnormal

1. **Cardiovascular system**: ( ) normal ( ) abnormal
2. **Respiratory evaluation:** ( ) normal ( ) abnormal
3. **Abdomen evaluation:**
4. Liver: ( ) normal ( ) abnormal
5. Spleen: ( ) normal ( ) abnormal
6. Other findings: _______________________________________________________________________________
7. Musculoskeletal: ( ) normal ( ) abnormal
8. **Laboratory exames - results**

________________________________________________________________________________________________

1. **Pediatric diagnosis:**
2. Feeding: ( ) Normal ( ) Inadequate
3. Nutritional diagnosis: ( ) Eutrophy ( ) Distrophy – describe: ______________________
4. Development: ( ) Normal ( ) Abnormal
5. Immunization: ( ) Adequate ( ) Inadequate

1. **DIAGNOSIS:** Congenital Zika Syndrome ( ) Exposure ( ) ZikV Negative ( ) ______________________________________________________________________
2. **MEDICAL CONDUCT**

_________________________________________________________________________________________

NAME AND SIGNATURE
