# Supplementary material for: Three-Year Clinical Follow-Up of Children Intrauterine Exposed to Zika Virus
Source: Viruses. 2021 Mar 22;13(3):523. doi: 10.3390/v13030523 (PMC8005078; doi:10.3390/v13030523)
Supplement: Supplementary file 1 [file viruses-13-00523-s001.zip › Attachment 3 CLINICAL MANIFESTATIONS OF CHILDREN EXPOSED TO ZIKV (1).docx]

| Attachment 3- CLINICAL MANIFESTATIONS OF CHILDREN EXPOSED TO ZIKV | | | | | | | | | | | | | | | | | | | | | | | | | | | | | | | | | | | | | | | | | | | | | | | | |  |  |
| --- | --- | --- | --- | --- | --- | --- | --- | --- | --- | --- | --- | --- | --- | --- | --- | --- | --- | --- | --- | --- | --- | --- | --- | --- | --- | --- | --- | --- | --- | --- | --- | --- | --- | --- | --- | --- | --- | --- | --- | --- | --- | --- | --- | --- | --- | --- | --- | --- | --- | --- |
|  |  |  | |  |  | | |  | | |  | | |  | | | | |  | | |  | | | | | | |  | | |  | | | |  | | | |  |  |  |  |  |  |  |  |  |  |  |
|  |  |  |  | |  |  | | | | | |  | | |  | | | | |  | | |  | | | | | | |  | | |  | | | |  | | | |  | | | | | | | |  |  |
| PREGNANT | | | | | **CHILD** | | | | | | | | | | | | | | | | | | | | | | | | | | | | | | | | | | | | | | | | | | | |  |  |
|  | | | | |  | | | | | | | | | | | | | | | | | | | | | | | | | | | | | | | | | | | | | | | | | | | |  |  |
|  | | | | |  | | | | | | | | | | | | | | | | | | | | | | | | | | | | | | | | | | | | | | | | | | | |  |  |
| CASE | **AGE** | **ETHNICITY** | **GESTATIONAL** | | **G.A(w)** | **SEX** | | | **W(g)** | | | | **L(cm)** | | | **HC (cm)** | | | | **MC** | | | | **APGAR**  **5 min** | | | **PCR ZIKV** | | | | **HEARING**  **LOSS** | | | **IMAGING** | | | | **D. A.** | | | | **OROPHARYNGAL**  **DYSPHAGIA** | | | | **LAST** | | |  |  |
|  |  |  | **USG** | |  |  |  |  |  |  |  |  |  |  |  |  |  |  |  |  |  |  |  |  |  |  |  |  |  |  |  |  |  |  |  |  |  |  | | | |  |  |  |  | **ASSESS. (m)** | | |  |  |
| 1 | 26 | M | 0 | | 39 | F | | | 2725 | | | | 48 | | | 34,5 | | | | 0 | | | | 10 | | | 0 | | | | 0 | | | 2 | | | | 0 | | | | 0 | | | | 3 | | |  |  |
| 2 | 22 | WH | 0 | | 38 +6 | F | | | 2800 | | | | 45 | | | 34 | | | | 0 | | | | 9 | | | 0 | | | | 0 | | | 2 | | | | 0 | | | | 0 | | | | 2 | | |  |  |
| 3 | 36 | WH | 0 | | 38+3 | M | | | 3255 | | | | 48 | | | 34,5 | | | | 0 | | | | 9 | | | 0 | | | | 0 | | | 0 | | | | 0 | | | | 0 | | | | 36 | | |  |  |
| 4 | 30 | B | 0 | | 37 | F | | | 3445 | | | | 50,5 | | | 33,5 | | | | 0 | | | | 9 | | | 0 | | | | 0 | | | 0 | | | | 0 | | | | 0 | | | | 36 | | |  |  |
|  |  |  |  |  |  |  |  |  |  |  |  |  |  |  |  |  |  |  |  |  |  |  |  |  |  |  |  |  |  |  |  |  |  |  |  |  |  |  |  |  |  |  |  |  |  |  |  |  |  |  |
| 5 | 14 | I | 0 | | 37+4 | M | | | 3250 | | | | 49 | | | 34 | | | | 0 | | | | 9 | | | 0 | | | | 0 | | | 2 | | | | 0 | | | | 0 | | | | 1 | | |  |  |
|  |  |  |  |  |  |  |  |  |  |  |  |  |  |  |  |  |  |  |  |  |  |  |  |  |  |  |  |  |  |  |  |  |  |  |  |  |  |  |  |  |  |  |  |  |  |  |  |  |  |  |
| 6 | 16 | WH | 0 | | 38+5 | M | | | 2860 | | | | 48 | | | 33 | | | | 0 | | | | 7 | | | 0 | | | | 0 | | | 2 | | | | 0 | | | | 0 | | | | at birth | | |  |  |
| 7 | 25 | WH | 0 | | 40+4 | F | | | 3040 | | | | 48,5 | | | 33 | | | | 0 | | | | 10 | | | 2 | | | | 0 | | | 2 | | | | 0 | | | | 0 | | | | 1 | | |  |  |
| 8 | 40 | WH | 0 | | 39+3 | F | | | 3160 | | | | 50 | | | 35 | | | | 0 | | | | 10 | | | 0 | | | | 0 | | | 2 | | | | 0 | | | | 0 | | | | at birth | | |  |  |
| 9 | 14 | B | 0 | | 38+5 | F | | | 3215 | | | | 47 | | | 33,5 | | | | 0 | | | | 9 | | | 0 | | | | 0 | | | 2 | | | | 0 | | | | 0 | | | | 1 | | |  |  |
| 10 | 28 | M | 0 | | 39+1 | F | | | 3775 | | | | 50,5 | | | 35,5 | | | | 0 | | | | 10 | | | 0 | | | | 0 | | | 0 | | | | 0 | | | | 0 | | | | 36 | | |  |  |
| 11 | 28 | M | 0 | | 39+4 | F | | | 3490 | | | | 48 | | | 33 | | | | 0 | | | | 9 | | | 2I | | | | 0 | | | 2 | | | | 0 | | | | 0 | | | | 36 | | |  |  |
| 12 | 29 | WH | 0 | | 38+1 | M | | | 3150 | | | | 49,5 | | | 33 | | | | 0 | | | | 9 | | | 0 | | | | 0 | | | delayed myelination | | | | autism spectrum disorder | | | | mild | | | | 36 | | |  |  |
| 13 | 39 | WH | 0 | | 35+4 | M | | | 2910 | | | | 48 | | | 34,5 | | | | 0 | | | | 9 | | | 0 | | | | 0 | | | 0 | | | | 0 | | | | 0 | | | | 26 | | |  |  |
| 14 | 31 | WH | 0 | | 40+1 | M | | | 3465 | | | | 48 | | | 35 | | | | 0 | | | | 10 | | | 0 | | | | 0 | | | 0 | | | | 0 | | | | 0 | | | | 36 | | |  |  |
| 15 | 15 | WH | 1 | | 39+4 | F | | | 3530 | | | | 52,5 | | | 37 | | | | 0 | | | | 10 | | | 0 | | | | 0 | | | 0 | | | | 0 | | | | 0 | | | | 32 | | |  |  |
| 16 | 34 | M | 0 | | 35+5 | F | | | 2270 | | | | 43 | | | 32,5 | | | | 0 | | | | 9 | | | 0 | | | | 0 | | | 0 | | | | 0 | | | | 0 | | | | at birth | | |  |  |
| 17 | 35 | M | 0 | | 35+5 | F | | | 2540 | | | | 46,5 | | | 33 | | | | 0 | | | | 9 | | | 0 | | | | 0 | | | 0 | | | | 0 | | | | 0 | | | | at birth | | |  |  |
| 18 | 36 | M | 0 | | 38+6 | M | | | 3595 | | | | 48 | | | 36,5 | | | | 0 | | | | 9 | | | 0 | | | | 0 | | | Parallelism of the lateral ventricles, supratentorial ectasia | | | | 0 | | | | 0 | | | | 36 | | |  |  |
|  |  |  |  |  |  |  |  |  |  |  |  |  |  |  |  |  |  |  |  |  |  |  |  |  |  |  |  |  |  |  |  |  |  |  | | | |  |  |  |  |  |  |  |  |  |  |  |  |  |
| 19 | 22 | WH | 0 | | 38+3 | M | | | 2615 | | | | 47,4 | | | 33 | | | | 0 | | | | 9 | | | 0 | | | | 2 | | | 2I | | | | 0 | | | | 0 | | | | at birth | | |  |  |
| 20 | 33 | M | 0 | | 38+6 | M | | | 3595 | | | | 50 | | | 36 | | | | 0 | | | | 10 | | | 0 | | | | 0 | | | 2 | | | | 0 | | | | 0 | | | | at birth | | |  |  |
| 21 | 38 | WH | 0 |  | 35+3 | | M | | | | 2665 | | | 46 | | | 32 | | | | 0 | | | | 10 | | | 0 | | | | 0 | | | | 0 | | | | 0 | | | 0 | | | | 19 | | | |
| 22 | 38 | WH | 0 |  | 35+3 | | M | | | | 2265 | | | 45 | | | 33 | | | | 0 | | | | 10 | | | 0 | | | | 0 | | | | 0 | | | | 0 | | | 0 | | | | 19 | | | |
| 23 | 14 | M | 0 |  | 40+1 | | M | | | | 3430 | | | 51 | | | 33 | | | | 0 | | | | 10 | | | 0 | | | | 0 | | | | 0 | | | | 0 | | | 0 | | | | 36 | | | |
| 24 | 23 | WH | 0 |  | 39+3 | | F | | | | 3250 | | | 48 | | | 34 | | | | 0 | | | | 9 | | | 0 | | | | 0 | | | | 0 | | | | 0 | | | 0 | | | | at birth | | | |
| 25 | 25 | WH | 0 |  | 34+1 | | M | | | | 2370 | | | 44 | | | 31 | | | | 0 | | | | 9 | | | 0 | | | | 0 | | | | 0 | | | | 0 | | | 0 | | | | 36 | | | |
| 26 | 17 | WH | 0 |  | 28 | | F | | | | 1270 | | | 38 | | | 27 | | | | 0 | | | | 9 | | | 0 | | | | 0 | | | | 0 | | | | 0 | | | 0 | | | | 29 | | | |
| 27 | 33 | WH | 0 |  | 39 | | F | | | | 3360 | | | 48 | | | 36 | | | | 0 | | | | 5 | | | 0 | | | | 0 | | | | 0 | | | | 0 | | | 0 | | | | 20 | | | |
| 28 | 34 | WH | 0 |  | 37 | | F | | | | 3600 | | | 48 | | | 34 | | | | 0 | | | | 9 | | | 0 | | | | 2 | | | | NI | | | | 0 | | | 0 | | | | 24 | | | |
| 29 | 15 | M | 0 |  | 37+4 | | F | | | | 2950 | | | 47 | | | 34,5 | | | | 0 | | | | 10 | | | 0 | | | | 0 | | | | 0 | | | | 0 | | | 0 | | | | 34 | | | |
| 30 | 22 | M | 0 |  | 38+6 | | M | | | | 3200 | | | 48,5 | | | 32 | | | | 0 | | | | 9 | | | 0 | | | | 0 | | | | 0 | | | | speech delay | | | 5 | | | | 36 | | | |
| 31 | 24 | WH | 1 |  | 37+3 | | M | | | | 3075 | | | 44 | | | 29,5 | | | | 1 | | | | 10 | | | 1 | | | | 0 | | | | Microcephaly brain calcifications, corpus callosum agenesis | | | | CZS | | | severe | | | | 34 | | | |
| 32 | 25 | WH | 0 |  | 39+1 | | F | | | | 4155 | | | 51,5 | | | 37 | | | | 0 | | | | 10 | | | 0 | | | | 0 | | | | 0 | | | | 0 | | | 0 | | | | 32 | | | |
| 33 | 37 | M | 0 |  | 39+4 | | F | | | | 3250 | | | 50 | | | 36 | | | | 0 | | | | 9 | | | 0 | | | | 0 | | | | 2 | | | | 0 | | | 2 | | | | at birth | | | |
| 34 | 32 | WH | 0 |  | 37+1 | | F | | | | 2620 | | | 48 | | | 33 | | | | 0 | | | | 9 | | | 2 | | | | 0 | | | | 2 | | | | 0 | | | 2 | | | | at birth | | | |
| 35 | 26 | WH | 0 |  | 38+6 | | M | | | | 2980 | | | 45 | | | 36 | | | | 0 | | | | 10 | | | 0 | | | | 0 | | | | 0 | | | | 0 | | | 0 | | | | 3 | | | |
| 36 | 18 | WH | 0 |  | 37+2 | | M | | | | 3230 | | | 50 | | | 35 | | | | 0 | | | | 9 | | | 0 | | | | 0 | | | | 0 | | | | speech delay | | | mild | | | | 36 | | | |
| 37 | **18** | **WH** | **1** |  | **39+2** | | **M** | | | | **3405** | | | **49,5** | | | **33** | | | | **0** | | | | **9** | | | **0** | | | | **0** | | | | **0** | | | | **0** | | | **4** | | | | **6** | | | |
| 38 | 30 | WH | 0 |  | 38+5 | | M | | | | 2855 | | | 49 | | | 33 | | | | 0 | | | | 7 | | | 0 | | | | 0 | | | | 2I | | | | 0 | | | 0 | | | | 3 | | | |
| 39 | 40 | M | 0 |  | 38+4 | | M | | | | 2750 | | | 47 | | | 31,5 | | | | 0 | | | | 9 | | | 0 | | | | 0 | | | | 0 | | | | cognitive delay, mild microcephaly | | | 0 | | | | 27 | | | |
| 40 | 25 | M | 0 |  | 39+2 | | F | | | | 3160 | | | 48 | | | 35 | | | | 0 | | | | 10 | | | 0 | | | | 0 | | | | 2 | | | | 0 | | | 2 | | | | at birth | | | |
| 41 | 30 | WH | 0 |  | 39 | | M | | | | 3525 | | | 52 | | | 36 | | | | 0 | | | | 9 | | | 0 | | | | 1 | | | | 0 | | | | 0 | | | 2 | | | | 30 | | | |
| 42 | 29 | M | 0 |  | 36+2 | | M | | | | 3055 | | | 49,5 | | | 35 | | | | 0 | | | |  | | | 2 | | | | 2I | | | | 2 | | | | 0 | | | 0 | | | | at birth | | | |
| 43 | **18** | **M** | **0** |  | **38+1** | | **M** | | | | **2580** | | | **47** | | | **31,5** | | | | **0** | | | | **9** | | | **0** | | | | **0** | | | | **0** | | | | **cognitive delay, mild microcephaly** | | | **4** | | | | **36** | | | |
| 44 | **35** | **M** | **0** |  | **38** | | **M** | | | | **2875** | | | **45,5** | | | **32,5** | | | | **0** | | | | **9** | | | **0** | | | | **0** | | | | **0** | | | | **0** | | | **mild** | | | | **18** | | | |
| 45 | 33 | WH | 0 |  | 38 | | F | | | | 3350 | | | 49 | | | 35,5 | | | | 0 | | | | 9 | | | 1 | | | | 0 | | | | 0 | | | | 0 | | | | 0 | | | 3 | | | |
| 46 | 20 | B | 0 |  | 40+1 | | M | | | | 2560 | | | 50 | | | | | 36 | | 0 | | | | 9 | | | 0 | | | | 0 | | | | 0 | | | | 0 | | | | 0 | | | 2 | | | |
| 47 | 30 | M | 0 |  | 34+4 | | | F | | 2200 | | | | 44,5 | | | | 32 | | | | 0 | | | | 9 | | | 0 | | | 0 | | | 2 | | | | 0 | | | | | | 0 | | | 2 | |  |
| 48 | 17 | M | 0 |  | 39+1 | | | F | | 2710 | | | | 45,5 | | | | 33,5 | | | | 0 | | | | 10 | | | 0 | | | 0 | | | 0 | | | | 0 | | | | | | 0 | | | 1 | |  |
| 49 | 20 | WH | 0 |  | 40+2 | | | M | | 3585 | | | | 52 | | | | 36,5 | | | | 0 | | | | 10 | | | 0 | | | NI | | | 2 | | | | 0 | | | | | | 0 | | | at birth | |  |
| 50 | 17 | M | 0 |  | 40 | | | F | | 3725 | | | | 50,5 | | | | 35 | | | | 0 | | | | 9 | | | 1 | | | 1 | | | 2 | | | | 0 | | | | | | 2 | | | 2 | |  |
| 51 | 21 | B | 0 |  | 39+4 | | | M | | 3360 | | | | 50 | | | | 36,5 | | | | 0 | | | | 9 | | | 0 | | | 0 | | | 0 | | | | 0 | | | | | | 0 | | | 1 | |  |
| 52 | 35 | WH | 0 |  | 39 | | | F | | 3345 | | | | 48 | | | | 35 | | | | 0 | | | | 10 | | | 1 | | | 0 | | | 2 | | | | 2 | | | | | | 2 | | | at birth | |  |
| 53 | 27 | NI | N/I |  | 40+1 | | | M | | 3320 | | | | 49,5 | | | | 35 | | | | 0 | | | | 10 | | | NI | | | 0 | | | 2I | | | | 0 | | | | | | 0 | | | at birth | |  |
| 54 | 27 | M | 0 |  | 40+1 | | | F | | 3500 | | | | 50 | | | | 33 | | | | 0 | | | | 9 | | | 0 | | | 0 | | | 0 | | | | 0 | | | | | | 2 | | | 11 | |  |
| 55 | 30 | WH | 0 |  | 39 | | | F | | 2650 | | | | 47 | | | | 33 | | | | 0 | | | | 10 | | | 0 | | | 0 | | | 0 | | | | 0 | | | | | | 0 | | | 3 | |  |
| 56 | 28 | WH | 1 |  | 36+5 | | | M | | 2695 | | | | 49 | | | | 33 | | | | 0 | | | | 9 | | | 0 | | | 0 | | | corpus callosum agenesis | | | | speech delay | | | | | | 0 | | | 18 | |  |

Subtitles: WH = White, B: Black, M: Brown, I = Indigenous, NI = No information; GA = gestational age in weeks; W= weight at birth in grams; L = length at birth in centimeters; HC = head circumference at birth in centimeters; MC = microcephaly; GESTATIONAL USG= ultrasonography during pregnancy compatible with ZIKV; IMAGE = alteration of postnatal image; D A= developmental abnormalities; LAST ASSESS. Age of the last assessment in months; 0= no adverse outcome; 1 = adverse outcome; 2= missing data; Bold= Zika and dengue coinfection in pregnant women
