# Supplementary material for: Three-Year Clinical Follow-Up of Children Intrauterine Exposed to Zika Virus
Source: Viruses. 2021 Mar 22;13(3):523. doi: 10.3390/v13030523 (PMC8005078; doi:10.3390/v13030523)
Supplement: Supplementary file 1 [file viruses-13-00523-s001.zip › Attachment 4 CLINICAL MANIFESTATIONS OF CHILDREN POSITIVE TO ZIKV (1).docx]

| Attachment 4-- CLINICAL MANIFESTATIONS OF CHILDREN POSITIVE TO ZIKV | | | | | | | | | | | | | | |
| --- | --- | --- | --- | --- | --- | --- | --- | --- | --- | --- | --- | --- | --- | --- |
| PREGNANT | | | | **CHILD** | | | | | | | | | | |
| CASE | **AGE** | **ETHNICITY** | **GESTATIONAL**  **USG** | **G.A(w)** | **SEX** | **W(g)** | **L(cm)** | **HC (cm)** | **APGAR**  **5 min** | **HEARING LOSS** | **IMAGING** | **D. A.** | **OROPHARYNGAL DYSPHAGIA** | **LAST**  **EVAL. (m)** |
|  |  |  |  |  |  |  |  |  |  |  |  |  |  |  |
| 1 | 30 | WH | 0 | 39+6 | F | 4065 | 49 | 35,5 | 10 | 0 | 2 | 0 | 0 | 6 |
| 2 | 22 | M | 0 | 38+5 | M | 4285 | 51 | 38 | 9 | 0 | ventricular dilation | cognitive delay | 0 | 10 |
| 3 | 15 | M | RCIU | 38+1 | M | 2600 | 49,5 | 31 | 9 | 1 | 2 | 0 | 0 | 32 |
| 4 | 17 | M | 0 | 38+5 | F | 2760 | 45,5 | 35 | 10 | 0 | 2 | S/I | 2 | at birth |
| 5 | 29 | WH | 0 | 38+6 | F | 3530 | 47 | 34 | 9 | 0 | 0 | 0 | 0 | at birth |
| 6 | 28 | WH | 0 | 40+5 | F | 3115 | 51 | 33,5 | 10 | 0 | 0 | 0 | 2 | at birth |
| 7 | 23 | M | 0 | 39+5 | M | 3780 | 52 | 34 | 9 | 0 | 2 | 0 | 0 | at birth |
| 8 | 34 | WH | 0 | 37+6 | F | 3750 | 46,5 | 34,5 | 9 | 0 | 2 | 0 | 2 | at birth |
|  |  |  |  |  |  |  |  |  |  |  |  |  |  |  |
| 9 | 30 | WH | 0 | 37 | M | 3745 | 48 | 36 | 9 | 0 | 0 | 0 | 0 | 19 |
| 10 | 24 | WH | microcephaly,  hydrocephaly,  brain calcifications | 37+3 | F | 3075 | 44 | 29,5 | 10 | 0 | microcephaly,  brain calcifications,  corpus callosum agenesis | microcephaly, severe dnpm delay, seizures | severe | 34 |
|  |  |  |  |  |  |  |  |  |  |  |  |  |  |  |
|  |  |  |  |  |  |  |  |  |  |  |  |  |  |  |
| 11 | 18 | WH | 0 | 38+5 | M | 3010 | 48 | 31,5 | 9 | 0 | 0 | 0 | 0 | 24 |
| 12 | 28 | M | 0 | 38+4 | F | 3915 | 48 | 37 | 9 | 0 | 0 | 0 | 0 | 20 |
| 13 | 38 | M | 0 | 38+6 | F | 2340 | 44,5 | 33 | 10 | 0 | 0 | TEA | 0 | 27 |
| 14 | 25 | WH | 0 | 41+1 | F | 3010 | 48 | 33,5 | 8 | 0 | 2 | 0 | 0 | 3 |
| 15 | 28 | M | 0 | 39+6 | F | 3375 | 50,3 | 36,5 | 10 | 0 | 2 | 0 | 2 | 3 |
| 16 | 30 | M | 0 | 39+4 | M | 3430 | 51 | 35,5 |  | 0 | 2 | 0 | 0 | 33 |
| 17 | 33 | WH | 0 | 38 | F | 3350 | 49 | 35,5 | 9 | 0 | 0 | 0 | 3 | 22 |
| 18 | 28 | WH | 0 | 38+3 | M | 2940 | 49 | 35 | 10 | 0 | 2 | 0 | 0 | at birth |
| 19 | 32 | WH | 0 | 39+6 | F | 4090 | 51 | 36 | 9 | 0 | 2 | 0 | 2 | 28 |
| 20 | 24 | B | 0 | 40 | M | 3290 | 51 | 35,5 | 9 | 0 | 0 | cognitive delay | mild | 30 |
| 21 | 17 | M | 0 | 40 | F | 3725 | 50,5 | 35 | 9 | 1 | 2 | 0 | 2 | at birth |
| 22 | 19 | WH | 0 | 40+6 | M | 3545 | 53 | 35 | 8 | 0 | 0 | 0 | 2 | 29 |
| 23 | 31 | WH | 0 | 39 | F | 3345 | 48,5 | 33,5 | 10 | 0 | 2 | 0 | 2 | 14 |
| 24 | 35 | WH | 0 | 39 | F | 3345 | 48 | 35 | 10 | 0 | 2 | 2 | 2 | at birth |
| 25 | 15 | M | 0 | 39+1 | M | 2800 | 47,5 | 32 | 10 | 0 | 2 | 0 | 0 | at birth |
| 26 | 21 | WH | 0 | 40+2 | F | 3650 | 49,5 | 33,5 | 9 | 0 | 2 | 0 | 2 | at birth |
| 27 | 38 | M | 0 | 32+6 | F | 1470 | 38 | 29 | 10 | 2 | 2 | 0 | 2 | at birth |

Subtitles: WH = White, B: Black, M: Brown; GA = gestational age in weeks; W= weight at birth in grams; L = length at birth in centimeters; HC = head circumference at birth in centimeters; MC = microcephaly; GESTATIONAL USG= ultrasonography during pregnancy compatible with ZIKV; IMAGE = alteration of postnatal image; D A= developmental abnormalities; LAST ASSESS. Age of the last assessment in months; 0= no adverse outcome; 1 = adverse outcome; 2= missing data; Bold= Zika and dengue coinfection in pregnant women
